# Bioinspired Collagen/κ-Carrageenan 3D Matrix for *In Vitro* Modeling of Vascular Calcification

**DOI:** 10.1021/acsbiomaterials.5c00754

**Published:** 2025-07-19

**Authors:** L. F. B. Nogueira, M. T. de Melo, J. G. Cominal, K. R. da Silva, S. Y. Fukada, M. Bottini, L. Brizuela, P. Ciancaglini, S. Mebarek, A. P. Ramos

**Affiliations:** † Department of Chemistry, Laboratory of Physical Chemistry of Surfaces and Colloids, Faculty of Philosophy, Science and Letters at Ribeirão Preto, 124588 University of São Paulo, 14040-901 Ribeirão Preto-SP, Brazil; ‡ Department of BioMolecular Sciences, School of Pharmaceutical Sciences of Ribeirao Preto, University of Sao Paulo, 14040-903 Ribeirão Preto-SP, Brazil; § Department of Experimental Medicine, 9318 University of Rome Tor Vergata, 00133 Rome, Italy; ∥ Sanford Burnham Prebys, La Jolla, California 92037, United States; ⊥ Université de Lyon, CNRS, UCBL, UMR 5246—Institut de Chimie et de Biochimie Moléculaires et Supramoléculaires, 43, Boulevard du 11 Novembre, 1918-69622 Villeurbanne, France

**Keywords:** vascular calcification, three-dimensional modeling, matrix mimicry, κ-carrageenan, cell transdifferentiation

## Abstract

Pathological calcification of soft tissue, particularly in vascular structures, is a hallmark of several cardiovascular diseases and significantly contributes to vascular stiffening and dysfunction. Despite sharing similarities with physiological ossification, the mechanisms driving the transdifferentiation of mouse vascular smooth muscle cells (MOVAS) into osteochondroblast-like phenotypes remain poorly understood. This transdifferentiation plays a critical role in the initiation and progression of pathological calcification. In this study, we developed a bioinspired 3D scaffold, composed of type I collagen (Col) and κ-carrageenan (κ-Carr), designed to mimic key aspects of the vascular extracellular matrix (ECM). This novel scaffold provides a physiologically relevant platform to study soft tissue calcification under osteogenic conditions. We demonstrated that this 3D system supports MOVAS cell adhesion, spreading, and transdifferentiation into a mineralizing phenotype in a controlled manner, as evidenced by the overexpression of osteogenic markers (TNAP and RUNX2), increased alkaline phosphatase activity, and controlled calcium phosphate deposition. Spectroscopic and thermogravimetric analyses revealed the formation of carbonated apatite minerals and a calcium-deficient apatite structure, indicative of controlled mineral deposition within the organic matrix. The incorporation of κ-carrageenan enhanced the calcification process, underscoring the importance of biochemical cues in directing the MOVAS phenotype changes. This scaffold system effectively replicates the spatial organization and physicochemical cues of the vascular ECM, providing a unique and innovative model to study pathological calcification processes. Moreover, this approach holds significant potential for developing regenerative biomaterials and therapeutic strategies aimed at preventing vascular calcification, opening new avenues for clinical applications and therapeutic interventions in cardiovascular disease.

## Graphical abstract

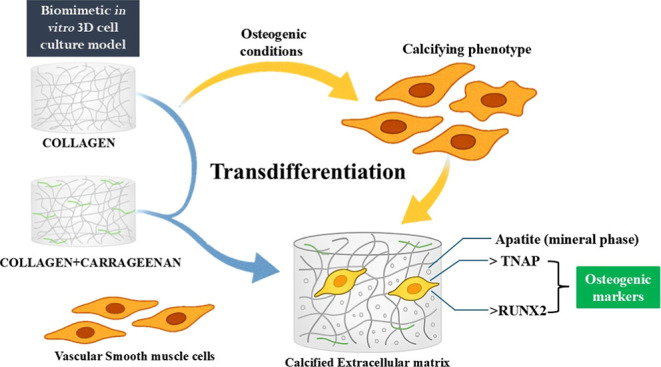

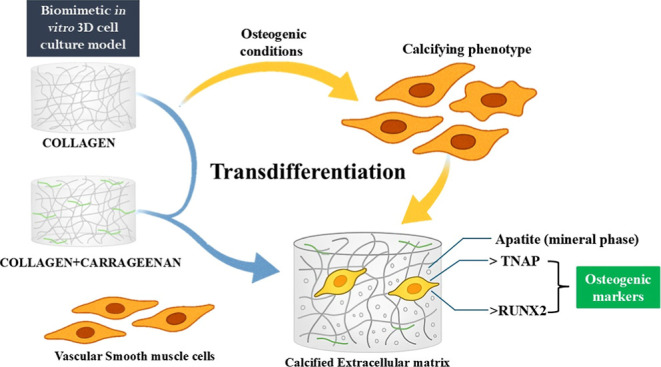

## 1 Introduction

One of the hallmarks of several pathologies such as diabetes, atherosclerosis, or chronic kidney disease is the development of calcifications in soft tissues, such as arteries and blood vessels.^1,2^ Pathological calcification formation is a dynamic process involving dysregulation of normal tissue homeostasis, influenced by inflammation and aging, leading to the deposition of calcium-containing minerals in soft tissues that are normally non-calcifying.^1,2^ Despite its high incidence, the detailed cellular mechanisms of calcification biogenesis remain elusive, considering metabolic imbalances and genetic conditions.^1,2^

Vascular calcification is a pathology associated with diseases like atherosclerosis, hypertension, aortic valve stenosis, coronary artery disease, diabetes mellitus, and chronic kidney disease.^3^ Described as the calcification of the medial vessel wall of arteries, it results in structural changes and the appearance of calcified plaques.^4,5^ Vascular calcification shares common features with endochondral-type ossification,^3,5,6^ with plaques consisting of carbonated apatite associated with a collagen-based organic matrix, which in turn leads to tissue injury and inflammation.^1,2^ At the cellular level, studies have revealed that resident cells in certain types of soft tissues, such as vascular smooth muscle cells (VSMCs), transdifferentiate toward osteochondroblastic-like cells, expressing bone-associated markers [e.g., tissue nonspecific alkaline phosphatase (TNAP) and runt-related transcription factor 2 (RUNX2)].^1,2,4–6^ This suggests that the formation of microcalcifications may be similar to physiological ossification processes.^1,2^ However, the genetic and epigenetic reprogramming involved in the transition of these cells from a contractile to a mineralizing state remains poorly explored.^7^

From a histological point of view, VSMCs play a crucial role in creating a microenvironment conducive to the deposition of apatite in the vessel wall during pathological calcification.^7,8^ In this context, to understand this process, the concept of a passive mechanism involving specific cellular signaling pathways and the reduction of circulating calcification inhibitors has been discussed, such as the hydrolysis of a constitutive mineralization inhibitor—inorganic pyrophosphate (PP_i_).^2,6,9,10^ However, it has been reported that cell transdifferentiation can be triggered by exogenous factors, including Ca^2+^ and inorganic phosphate (P_i_) imbalance, a condition mainly found in chronic kidney disease.^7,8^ In this scenario, it has been demonstrated that VSMCs undergo differentiation toward osteogenic cells when exposed to a P_i_ concentration of around 2.6 mmol L^−1^.^7,11,12^

Although studies on the genetic reprogramming of VSMCs to acquire characteristics similar to those of osteoblasts have been reported, this remains an area of active investigation.^7,13^ Given the incomplete understanding of the cellular and molecular mechanisms during this transdifferentiating process and the surrounding environment, treatment options remain limited.^3,4,14,15^ In this sense, advanced understanding of the cell behavior under exogenous mineralizing conditions is crucial for comprehending not only natural bone formation but also for preventing and treating pathological calcification.^3,4,9,10,15,16^

To achieve this, it is essential to emphasize the influence of the extracellular matrix (ECM) structure and composition on VSMC activity.^17–19^ The ECM, beyond providing mechanical support, regulates cellular functions such as differentiation, migration, growth, survival, and maturation of cells into a calcifying phenotype.^17–19^ Its properties facilitate biochemical and biomechanical signals, essential for morphogenesis, homeostasis, and tissue formation through various signaling pathways.^17^ The microenvironment is critical, since surrounding cells constantly sense cell–matrix interactions, thus influencing decisions about their fate.^18–20^ Therefore, VSMCs must attach to a controlled microenvironment, which must mediate both the complex cell−cell and cell–matrix interactions that occur in vivo to undergo genetic reprogramming based on epigenetic changes.^3–5,14,15,18,19^

2D in vitro models are commonly used to investigate both physiological and pathological calcification processes. Despite their significant contribution, 2D cultures may induce a flattened morphology, altering cytoskeletal organization and gene expression, potentially impacting cell differentiation pathways.^18,19,21^ In response to these limitations, the development of 3D models becomes paramount, allowing cells to maintain their natural shape, to keep genotypes, and to interact with the environment in 3D, offering a reliable approximation to the in vivo microenvironment.^18,19,21^ Although mimicking the natural ECM microenvironment in vitro is challenging, the development of synthetic models that faithfully reproduce the structure of calcified tissue has expanded possibilities to understand cellular responses with precision and reproducibility.^5,18,21,22^

The ECM of connective tissues is primarily composed of fibrous structures.^23^ Pathologies associated with connective tissue are often associated with changes in ECM structure and composition.^23^ Although collagen is the most abundant protein in mammalian tissues, other noncollagenous macromolecules, such as glycosaminoglycans (GAGs), play key functional roles in the ECM, as already reported in the literature.^21,23,24^ For instance, dermatan sulfate, present in vessels, plays a crucial role in physiological functions through interactions with collagens, growth factors, and heparin cofactor-II.^25^ The function of these compounds in tissue structure and stimulation of cell activity should be explored in developing 3D scaffolds aiming at replicating the ECM in both physiological and pathological conditions.^26–28^

Given the need for predictive in vitro models that replicate the complexity of vascular tissue remodeling, we developed a 3D scaffold composed of type I collagen (Col) and κ-carrageenan (κ-Carr), a sulfated polysaccharide structurally analogous to GAGs and derived from renewable sources.^29–31^ This bioinspired scaffold was designed to mimic key structural and compositional features of the vascular ECM, thereby providing relevant biochemical and topographical cues.^29–31^ Building on our previous work with Col/κ-Carr matrices,^24^ we hypothesized that this system could support mouse vascular smooth muscle cells (MOVAS) adhesion, proliferation, and transdifferentiation into an osteochondroblast-like phenotype under osteogenic stimulation. This study aims to establish a proof-of-concept 3D collagen/κ-carrageenan scaffold model to investigate osteogenic modulation of VSMCs in vitro. To this end, we evaluated the effect of this sulfated polysaccharide on the composition of the scaffolds and the resulting influence on mineralization by MOVAS cells, aiming to establish a robust platform for modeling soft tissue calcification. In the future, this model may serve as a valuable tool for mechanistic studies, regenerative biomaterial design, and the development of miniaturized assays for screening therapeutic strategies targeting pathological mineralization.

## 2 Materials and Methods

### 2.1 Collagen Extraction and Purification

Based on the procedure reported in our previous work,^24^ type I collagen (Col) was isolated from rat tail tendons kindly provided by the vivarium of the *Hemocentro de Ribeirão Preto—University of São Paulo, Brazil*. These tendons were obtained from animals that had been previously euthanized for routine colony management or unrelated approved procedures. The tails were considered biological surplus and would have otherwise been discarded as part of the vivarium’s routine operations. No animals were sacrificed or subjected to any procedures specifically for this study.

The collection and use of this material were carried out in direct collaboration with members of the Animal Ethics Committee (CEUA) of the Ribeirão Preto Medical School (FMRP-USP), which is fully accredited and operates in accordance with the guidelines of the Brazilian National Council for the Control of Animal Experimentation (CONCEA). As the material originated from nonexperimental surplus and no procedures were performed on the animals for the purposes of this study, a formal experimental protocol number was not issued. Nonetheless, all procedures adhered to institutional ethical principles and were conducted under CEUA-FMRP oversight for the responsible use of animal-derived biological materials in research.

The purity of the Col solution was evaluated by SDS-PAGE electrophoresis under denaturing conditions, using 7 wt % polyacrylamide gel and silver nitrate for protein band staining. The migration pattern of the sample was compared to the Thermo Scientific PageRuler Plus Prestained Protein Ladder, which consists of a mixture of nine recombinant proteins with molecular weights ranging from 10 to 250 kDa. The conformation of Col molecules in a solution containing 0.5 mol·L^−1^ acetic acid and 5 μmol L^−1^ of protein was evaluated by circular dichroism spectroscopy on a JASCO 810 spectrophotometer. The results of the collagen solution characterizations were previously reported in our earlier study.^24^ Afterward, the purified Col solution was concentrated using a tangential filtration system (Labscale), containing a Pellicon cassette with a 5 kDa cutoff membrane (Millipore), resulting in a final concentration around 10 mg·mL^−1^.

### 2.2 Preparation of Col-Based Scaffolds

Based on this final concentration, 3D scaffolds were prepared by the addition of approximately 4 mL of this solution into a 3.3 cm^3^ cylindrical Teflon template. Then, the template was exposed to an atmosphere of NH_3(g)_, generated from the decomposition of (NH_4_)_2_CO_3_, in a closed box, for 24 h.^24^ The exposure of highly concentrated collagen to ammonium vapor promotes fibrillogenesis in vitro.^32–34^ After this process, the hydrogel obtained from the aggregation of microfibrils was dehydrated in a climatic chamber with controlled humidity at 40% and temperature at 37 °C, promoting a reduction of the volume to obtain a dense fibrillar matrix composed of Col.

As described in our previous work,^24^ the incorporation of κ-Carr was carried out by adding the polysaccharide to the Col solution at a final concentration of 5 wt % relative to a Col concentration of 40 mg·mL^−1^.^35–38^ This percentage was chosen based on the compositions of blood vessels reported in the literature.^39^ After obtaining the scaffolds, designated as S­(Col) and S­(Col + 5% κ), respectively, the samples were characterized before and after cell cultures by microscopy and spectroscopy techniques.

### 2.3 Cell Cultures in the Presence of the Scaffolds

The commercial MOVAS line (ATCC CRL-2797) was used in the experiments aiming at reproducing VSMCs transdifferentiation toward calcifying cells. The cells were cultured in growing medium containing high glucose (4.5 g·L^−1^) DMEM (Dulbecco’s Modified Eagle Medium, Sigma-Aldrich) with 10 vol % Fetal Bovine Serum (FBS, Gibco), 100 U·mL^−1^ penicillin, and 100 μg·mL^−1^ streptomycin. When cells reached around 80% confluence, they were harvested using 0.05% trypsin/ethylenediaminetetraacetic acid (EDTA) (Sigma-Aldrich) and seeded in the scaffolds under exogenous supplementation with 0.28 mmol L^−1^ ascorbic acid (AA) and 10 mmol L^−1^ β-glycerophosphate (β-GP). AA and β-GP are two osteogenic factors commonly used to stimulate osteogenic differentiation and mineralization of MOVAS cells.^5,40^ Culture time was chosen according to the aim.

Then, MOVAS were seeded at a density of 40,000 cells per Col-based scaffolds, S­(Col) and S­(Col + 5% κ), which were previously placed at the bottom of the 96-well culture plate and preconditioned with culture medium 24 h prior. Cells at the same density (40,000 cells per cm^2^) were seeded in the sterilized 96-well culture plates and cultured in 2D as control. The plates containing the cells were incubated in a humidified atmosphere consisting of 95% air and 5% CO_2_ at 37 °C. The evolution of the cell culture over the days, in both 2D and 3D, was followed by optical microscopy. The images were acquired using a transmission light microscope (Nikon Eclipse), and contrast/brightness adjustments were applied uniformly across all images using ImageJ software.

### 2.4 Cell Viability (MTT Assay)

The tetrazolium-based colorimetric assay (MTT) was performed at day 7 of culture for MOVAS to assess cell survival and proliferation in both the absence and presence of the scaffolds. This colorimetric assay method is based on the transformation of yellow MTT (3-[4,5-dimethylthiazol-2-yl]-2,5-diphenyltetrazolium bromide) into purple formazan crystals by viable and metabolically active cells.^41^ MTT labeling reagent (0.125 mg·mL^−1^ final concentration) was added to each well, and the cells were further incubated for 4 h. Then, the supernatant was replaced by 1 mL of dimethyl sulfoxide (DMSO, Sigma-Aldrich). Cell viability was directly related to absorbance measured at 570 nm using a Tecan Infinite M200 microplate reader. The results were presented as metabolic activity values normalized to the absorbance of the 2D control condition, which was set to 100%. Therefore, values higher than 100% indicate a relative increase in metabolic activity rather than absolute viability.

### 2.5 Characterization of Cell Morphology by Microscopic Analysis

For confocal microscopy, on the 21st day of MOVAS cultivation, the scaffolds were washed twice with phosphate-buffered saline (PBS) and fixed with 2% (v/v) paraformaldehyde diluted in PBS for 20 min on ice. Then, the scaffolds were washed five times with PBS containing 100 mmol L^−1^ glycine (Sigma-Aldrich) and subsequently permeabilized with 0.1% (v/v) Triton X-100 (Sigma-Aldrich) for 10 min at room temperature. After being washed, cells were incubated with Alexa Fluor 488 dye (1:500, Invitrogen), a green-fluorescent dye with excitation designed for use with the 488 nm laser line, for 40 min at room temperature. Later, scaffolds were washed five times in PBS, rinsed quickly with distilled water, and mounted onto glass slides using ProLong Diamond antifade reagent mounting medium (Life Technologies). Cells were imaged at 63× magnification by a laser scanning probe confocal microscope (Leica TCS SP8). For F-actin visualization, cells were stained with Alexa Fluor 568-conjugated phalloidin (diluted 1:500 in PBS, Invitrogen) for 10 min at room temperature. For nuclear localization studies, nuclei were stained with DAPI (4′,6-diamidino-2-phenylindole, Invitrogen) at a final concentration of 1 μg·mL^−1^ for 5 min. Phalloidin was detected at 488 and 518 nm, and DAPI was detected at 405 and 461 nm, respectively.

Additionally, scanning electron microscopy (SEM) was used for the morphological analysis of MOVAS cultured for 21 days in the presence of both organic matrix compositions, S­(Col) and S­(Col + 5% κ), along with exogenous osteogenic supplementation. Based on an osmium tetroxide post fixed scaffold, the positive staining for osmium indicated the presence of the lipid membrane, allowing for excellent preservation of fine features. For this, scaffolds were washed twice with PBS (pH 7.4) and fixed with 2% (v/v) paraformaldehyde diluted in PBS for 1 h on ice and then washed with PBS. Subsequently, scaffolds were postfixed with 1% (w/v) OsO_4_ in PBS for 1 h. Then, the samples were systematically dehydrated in a graded series of ethanol (from 50% (v/v) to 100% (v/v)) at room temperature.

### 2.6 Calcium Nodule Detection Using Alizarin Red Staining Assay

On the 21st day of the MOVAS culture, the medium was removed from the wells of the 96-well culture plate, and the scaffolds containing these cells, which were kept at the bottom of each well, were washed with PBS five times. After, the scaffolds were incubated with 2% (w/v) alizarin red solution in PBS (pH 5.0) for 20 min at room temperature. The stained scaffolds were washed three times with deionized water to remove the excess dye. As described by Bougault et al.,^42^ quantification of staining was conducted using 100 mmol L^−1^ cetylpyridinium chloride pH 7 (CPC, Sigma-Aldrich) and absorbance was measured at 570 nm. The results were expressed as a percentage relative to control (cells seeded in 2D, the polystyrene coverslip).

Due to the known nonspecific binding of Alizarin Red S to collagen scaffolds, which results in intense background staining, no representative images are shown for the 3D conditions [S­(Col) and S­(Col +5% κ)]. To account for this interference, absorbance values obtained for mineralized scaffolds were corrected by subtracting baseline absorbance values measured from stained but nonmineralized scaffolds of identical composition. This approach, adapted from Zhang et al.,^43^ allowed for accurate quantification of calcium deposition while minimizing interference from nonspecific dye adsorption by the matrix.

### 2.7 Total Calcium Concentration and Protein Dosage

As described by Borel et al.,^44^ total calcium concentration was measured by a colorimetric assay, using *o*-cresolphthalein complexone method (Sigma-Aldrich). For this, after washing with PBS, deposits containing calcium formed after culturing MOVAS for 21 days in the presence of osteogenic factors were isolated from the organic matrix using 200 μL of 0.6 mol·L^−1^ HCl (Synth) and kept overnight at room temperature. Then, the supernatants were collected. After decalcification, scaffolds were washed three times with PBS, solubilized with 0.1 mol·L^−1^ NaOH (Synth), containing 0.1 wt % sodium dodecyl sulfate (SDS, Sigma-Aldrich), and the collected supernatants were incubated for 5 min with 2-amino-2-methyl-1-propanol (Sigma-Aldrich) and *o*-cresolphthalein complexone reagent (Sigma-Aldrich). Absorbance was measured at 570 nm by a TECAN Infinite M200 Pro microplate reader. The total amount of calcium was normalized by the protein amount in the samples.

For protein dosage, the scaffolds containing cells were disrupted by sonication, then centrifuged for 5 min at 2000*g*, at 4 °C. Then, 10 μL of supernatant was added to 200 μL of a Bi-Cinchoninic Acid (BCA) Protein Assay (Thermo Scientific). The mixture was incubated for 30 min at 37 °C. The reaction was stopped by thermal shock (plunging into ice). Absorbance was measured at 562 nm by a TECAN Infinite M200 Pro microplate reader.^5,44^ A calibration curve was obtained using bovine serum albumin (BSA) as the standard.

### 2.8 Tissue Non-specific Alkaline Phosphatase (TNAP) Activity Measurements

For determination of TNAP activity based on the amount of *p*-nitrophenol (pNP) released after dephosphorylation of *p*-nitrophenyl phosphate (pNPP), adapting from the literature,^5,45,46^ cells were collected in 0.2 wt % NP-40 Protein detergent (Thermo Scientific) and disrupted by intense sonication for 20 s. Then, cell lysates were centrifuged (2000*g*, 5 min, 4 °C) and the obtained supernatants were used to determine TNAP activity. 10 μL of these supernatants was preincubated for 5 min at 37 °C. Then, 190 μL of a mixture containing 10 mmol L^−1^ p-NPP as a substrate at pH 10.4 (Sigma-aldrich), 0.56 mmol L^−1^ 2-amino-2-methyl-1-propanol (Sigma-aldrich), and 1 mmol L^−1^ MgCl_2_ (Synth) was added to each supernatant. The rate of pNP formation was measured by reading the absorbance at 405 nm, for 2 min every 10 s, by a TECAN Infinite M200 Pro spectrophotometer. Specific activity was expressed as μmol of pNP released per min and per milligram of protein (μmol·min^−1^·mg^−1^ or U·mg^−1^). Protein concentration was determined as previously described.

### 2.9 Western Blot

MOVAS cells cultured on scaffolds were lysed in ice-cold RIPA buffer (RIPA; 25 mmol L^−1^ Tris-HCl, 150 mmol L^−1^ NaCl, 1% NP-40, 1% sodium deoxycholate, 0.1% SDS, pH 7.4) for 30 min. Total protein concentration in lysates was determined using a BCA protein assay kit (Thermo Scientific), and equal amounts of protein (20 μg per lane) were diluted in Laemmli buffer (Bio-Rad, California, USA), boiled for 5 min, and loaded onto 10% SDS−polyacrylamide gels.

Electrophoresis was performed alongside PageRuler Plus Protein Ladders (Thermo Scientific), followed by protein transfer to nitrocellulose membranes (GE Healthcare). Transfer efficiency and protein loading were confirmed by Ponceau S staining. After the transfer, membranes were blocked in 5% (w/v) BSA in TBS-T (Tri*sec*-buffered saline containing 0.1% Tween 20 detergent) for 1 h at room temperature. Then, these membranes were incubated with primary rabbit monoclonal anti-TNAP (diluted 1:500 in TBS-T, Invitrogen) and anti-RUNX2 (diluted 1:500 in TBS-T, Invitrogen) overnight at 4 °C with gentle rocking. Membranes were then washed with TBS-T and probed with goat antirabbit and horseradish peroxidase (HRP)-coupled antibodies (Thermo Scientific) for 1 h at room temperature. Immunoblots were revealed with the enhanced chemiluminescence (ECL) detection system (Bio-Rad), and images were captured with the XRS+ Chemidoc (Bio-Rad) camera.

The intensity of the bands was quantified using ImageJ software and normalized to the 2D control condition (set to 1).^44,47^ One technical duplicate was included in the TNAP blot to confirm reproducibility. An independent biological replicate was also performed and is included in the Supporting Information, along with full-length uncropped membranes and the Ponceau S-stained image (Figures S1 and S2).

### 2.10 Thermogravimetric Analysis (TGA)

To quantify the formation of minerals in the samples, TGA analyses were conducted using the SDT-Q600 DSC-TGA instrument (TA Instrument), at a heating rate of 5 °C·min^−1^ within the temperature range of 20−900 °C under an air flow rate of 100 mL·min^−1^. The scaffolds before and after cell culture were standardized in size and shape. The mineral content was calculated from the difference in the weight of the scaffolds before and after the cell culture, combusted at 600 °C, since at this temperature the organic portion of the samples should be completely combusted in an oxygen-containing environment.^48^

### 2.11 Compositional Characterizations by Spectroscopic Techniques: FTIR, Raman, and EDS

The mineralization of the scaffolds was also evaluated by Fourier-transform infrared spectroscopy (FTIR), Raman spectroscopy, and Ca/P molar ratio calculated from X-ray energy-dispersive (EDS) analysis. For this, on the last day of cell culture, the medium was removed, and the scaffolds were washed with PBS (pH 7.4) and fixed with 2% paraformaldehyde diluted in PBS for 20 min on an ice bath. Next, the samples were washed with PBS and systematically dehydrated in a graded series of ethanol (from 30% to 100%), at room temperature.

### 2.12 Statistical Analyses

All quantitative measurements were carried out at least in triplicate. All the data were expressed as mean ± standard deviation (SD). To establish the significance of our results, data were analyzed using a two-way analysis of variance (ANOVA), assuming a confidence level of 95% (*p* < 0.0001) for statistical significance. Graphs and calculations were done using Origin (OriginLab) and Prism (GraphPad software).

## 3 Results and Discussion

### 3.1 Microscopic Characterization and the Influence of the Biomimetic ECM on MOVAS Spreading and Proliferation

The effects of 2D and 3D biomimetic matrices on MOVAS seeding and maturation were evaluated by using optical microscopy (Figure 1). Brightness and contrast were adjusted digitally to enhance the visualization of cellular distribution and scaffold architecture. The analysis of the optical micrographs obtained for MOVAS seeded in 2D over time (Figure 1C,F,I) revealed changes in cell morphology, characterized by an increase in cell size and volume after 21 days of culture. These features suggest that hypertrophy may be associated with the phenotypic transition of MOVAS toward osteoblast-like cells. Although the precise molecular signaling involved in MOVAS transdifferentiation was not dissected in this study, the observed morphological changes and mineralization patterns support the relevance of this 3D model as a foundation for future mechanistic and pharmacological investigations.

**Figure 1:**
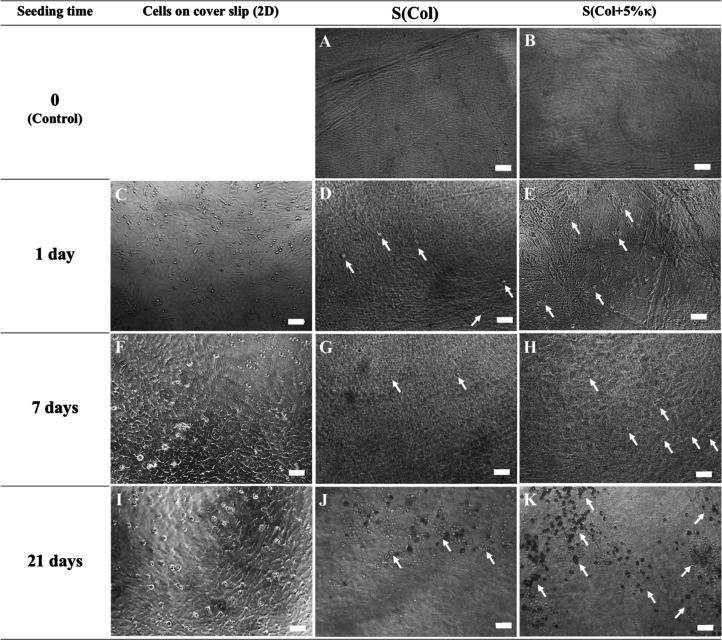
Morphology of MOVAS cultured in a 2D and 3D *in vitro* microenvironment. Representative transmission light microscopy images of MOVAS cells seeded on polystyrene coverslip (2D, micrographs in (C,F,I)) and collagen-based scaffolds: S­(Col) [micrographs in (A,D,G,J)] and S­(Col +5% κ) [micrographs in (B,E,H,K)]. Images were acquired using transmission light microscopy at days 1, 7, and 21 of culture. Brightness and contrast were digitally adjusted postacquisition to improve visualization of cell distribution and scaffold architecture. Arrows indicate regions of cell aggregation and interaction with scaffold fibers. Bar length: 100 μm.

Cells may undergo osteoprogenitor transformation from exogenous stimuli, leading to phenotypic changes.^18,19^ During this process, the volume of hypertrophic cells increases, and the extracellular environment becomes mineralized to develop calcified tissue. The stimulation of calcium deposition by osteoprogenitor cells depends on exogenous supplements, such as AA and β-GP, which induce slow and dispersed mineral nodules formation across the ECM, differing from the coordinated mineralization of calcified tissues in vivo.^18,19,49^

Micrographs obtained after 1 day of seeding reveal changes in the surface morphology of 3D scaffolds, which seem to become rougher after cell cultivation (compare Figure 1A,B with [Fig fig1]D,E). Moreover, the presence of cells can be characterized by micrometric structures with a circular shape as observed in 2D (white arrows in Figures 1C–E). Different morphological changes were observed during the cultivation of MOVAS cells over time, suggesting distinct responses to the microenvironment (compare Figure 1G,H with [Fig fig1]J,K). An increase in the number of cells at the surface can be visualized in MOVAS-seeded scaffolds, especially in the presence of κ-Carr (Figure 1H,K).

Scaffold cytotoxicity was assessed using the MTT assay, and no cytotoxic effects were observed compared with 2D cultures. Notably, the increase in metabolic activity after seeding on the 3D biomimetic scaffolds, particularly those containing κ-Carr, suggests a higher number of metabolically active cells and enhanced mitochondrial function in MOVAS cultures (Figure 2). This observation is consistent with previous studies, such as Cao et al.,^50^ which report the stimulatory effect of κ-Carr on cellular activities, attributed to its sulfate groups that mimic the charge and structural function of native ECM components (Figure 2).

**Figure 2:**
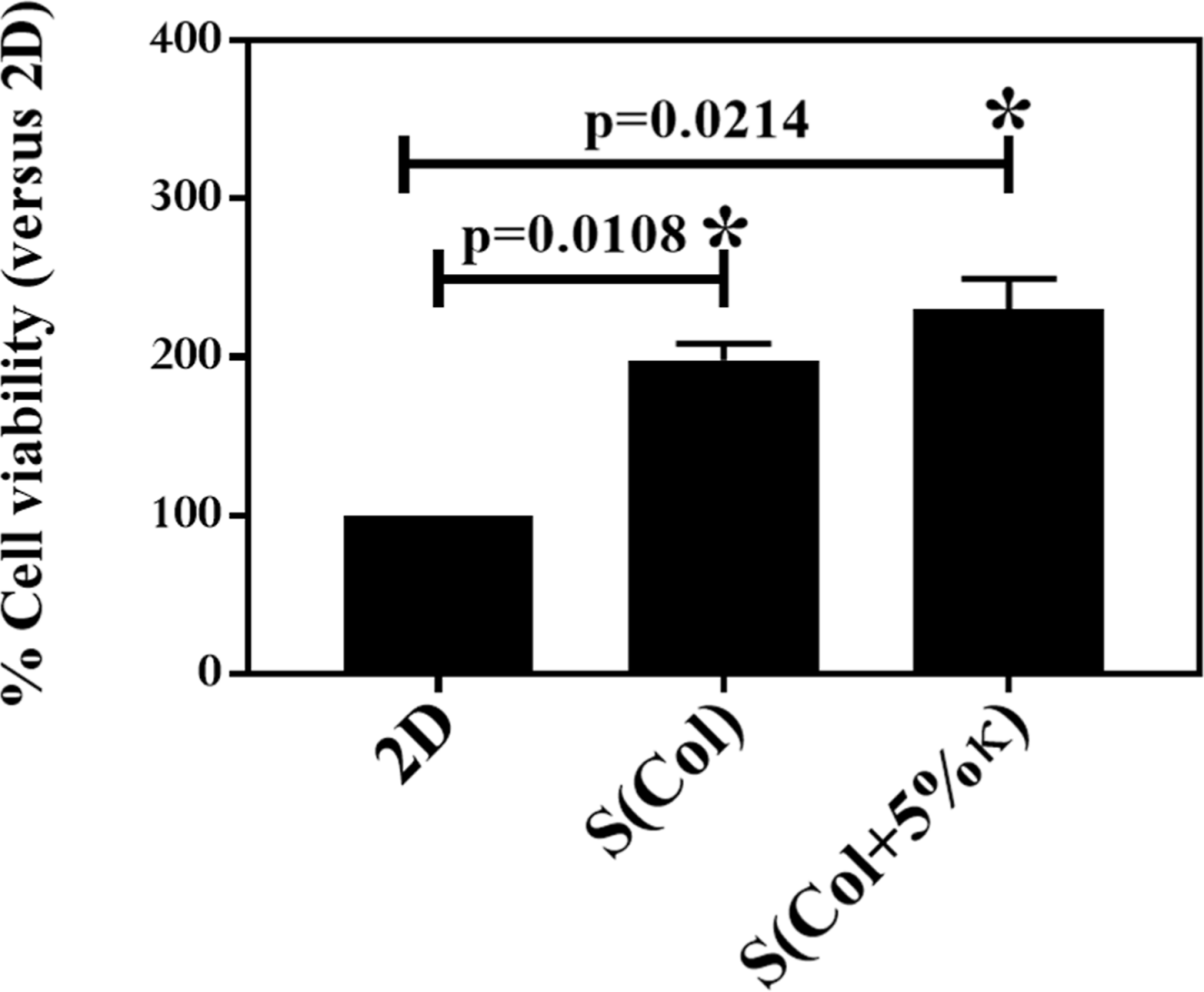
Effect of the scaffold’s composition on the viability of MOVAS. Metabolic activity of MOVAS cells cultured for 7 days on 2D substrates (control), Col-based scaffolds [S­(Col)], and scaffolds containing 5 wt % κ-Carr [S­(Col +5% κ)]. MTT assay results are expressed as absorbance values normalized to the 2D control (set to 100%), representing relative metabolic activity. Data are reported as mean ± standard deviation calculated on three independent samples, each performed in triplicate. Statistical analysis was performed using two-way ANOVA with Tukey’s post hoc test; *p < 0.05.

Examining cell spreading and cytoskeletal organization through phalloidin and DAPI staining, confocal images (Figure 3) revealed a 3D dispersion of nuclei within the scaffold structure, suggesting a favorable microenvironment for cell proliferation and spatial organization. The cytoskeleton plays a key role in regulating migration, adhesion, and cellular shape.^51^ In both scaffold conditions (i.e, in the presence and absence of κ-Carr), actin filaments were distributed along the porous matrix, supporting cell−scaffold anchoring.

**Figure 3:**
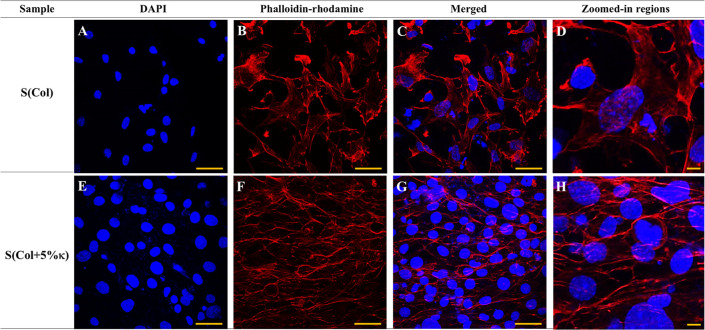
Confocal laser scanning microscopy images showing the effect of biomimetic ECM composition on the MOVAS morphology under osteogenic stimulation. MOVAS cells were cultured for 7 days on Col-based scaffolds without [(A–D) S­(Col)] or with 5 wt % κ-Carr [(E–H) S­(Col+5%κ)]. Cytoskeletons were stained with phalloidin−rhodamine (red) and nuclei were stained with DAPI (blue). Zoomed-in panels (D,H) correspond to representative regions selected from (C,G), respectively, and highlight actin organization and cell–matrix interactions in greater detail. Scale bars: 20 μm (A–D,E–H); 10 μm (D,H).

To improve visualization, enlarged images of specific areas highlighting cytoplasmic projections and actin-rich extensions that span scaffold pores were included (Figure 3D,H). In S­(Col) (Figure 3A–D), cells exhibited elongated morphologies and directional cytoskeletal arrangements, while in S­(Col +5% κ) (Figure 3E–H), cells exhibited higher density, more aligned actin bundles, and increased nuclear density. These qualitative features support the role of κ-Carr in enhancing cell–matrix interactions and promoting scaffold remodeling. κ-Carr has previously been associated with increased cell adhesion, spreading, and proliferation.^50^ No morphological evidence of apoptosis was observed in either of the conditions.

Although quantitative morphometric analysis (e.g., circularity or aspect ratio) was not performed in this study, the observed cytoskeletal organization and nuclear distribution are consistent with remodeling processes induced by osteogenic stimuli.

Taken together, these findings highlight the potential of this bioinspired scaffold as a simple yet robust in vitro platform to investigate the MOVAS phenotypic modulation and mineralization, offering an accessible complement to more complex organotypic or animal models. In this context, the biochemical stimulus of a 3D biomimetic ECM, combined with osteogenic factors such as ascorbic acid (AA) and β-glycerophosphate (β-GP), may promote the transdifferentiation of MOVAS cells toward a calcifying osteochondroblast-like phenotype. So, this process can be evaluated through the secretion of ECM components and calcification of the organic matrix.

Scanning electron microscopy (SEM) (Figure 4) was employed to investigate the morphological features of the scaffold surfaces before and after 21 days of MOVAS culture. Prior to cell seeding (Figure 4A), the collagen-based scaffolds [S­(Col)] exhibited a dense and organized network of compacted, intertwined fibrils arranged in a parallel pattern, consistent with characterization carried out in a previous study.^24^ The incorporation of 5 wt % κ-Carr did not significantly disrupt this fibrillar organization, as a similar dense architecture was maintained in S­(Col +5% κ) (Figure 4B), similar to a previous report.^24^

**Figure 4:**
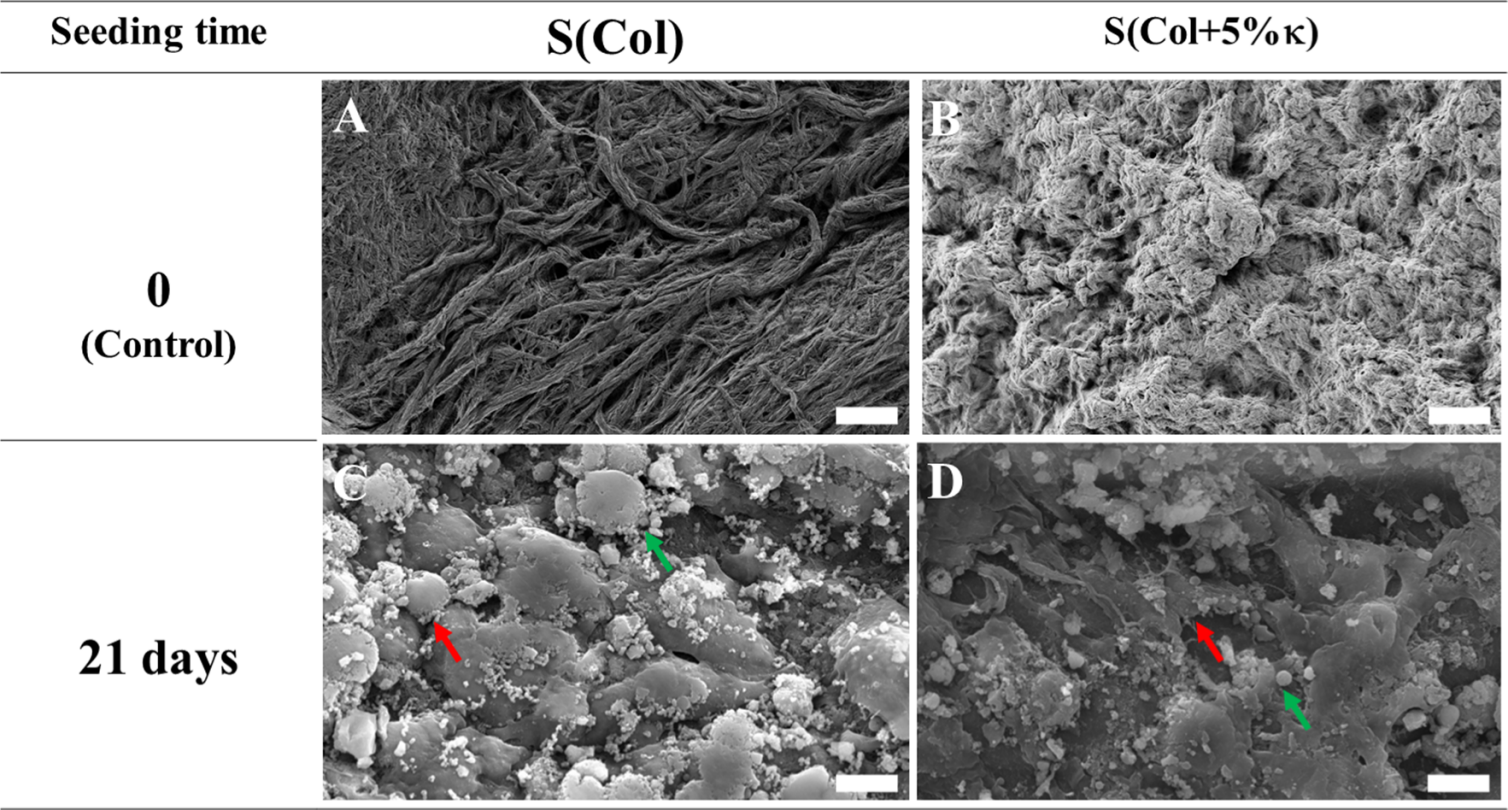
Morphological characterization of Col-based scaffolds. Scanning electron microscopy images of Col-based scaffolds before (A) and after addition of 5 wt % κ-Carr (B). Electron micrograph of MOVAS cultured for 21 days with osteogenic factors, respectively, in Col-based scaffolds fabricated without (C) and with the addition of 5 wt % κ-Carr (D). Red and green arrows indicate the presence of cells adhered to the scaffolds (C) and microsized spheroidal structures close to cellular filopodia (D), respectively. Bar length: 10 μm.

After 21 days of culture under osteogenic conditions, SEM images revealed cells adhered to the scaffold surfaces (red arrows, Figure 4C,D). Notably, cells on S­(Col +5% κ) (Figure 4D) appeared more elongated than those on S­(Col) (Figure 4C), suggesting that κ-Carr may enhance cytoskeletal extension and directional growth. These qualitative observations align with confocal microscopy findings (Figure 3), suggesting that the presence of this polysaccharide creates a favorable microenvironment that promotes spindle-shaped morphology and supports essential cellular functions.^18,19,21,50^ This may indicate a potential maximization of the cell-biomimetic ECM interaction. Given the critical role of morphology in processes such as migration and shape maintenance, the incorporation of κ-Carr in 3D model development may help preserve the cells’ native shape, minimize genotype alterations, and enhance their ability to interact with the surrounding matrix, thereby providing a more physiologically relevant approximation of the in vivo microenvironment.^18,19,21,50^ While quantitative morphometric analyses (e.g., elongation index and surface coverage) were not performed in this study, their inclusion is acknowledged as a valuable direction for future refinement. Nonetheless, the Col/κ-Carr scaffold constitutes a reproducible and adaptable platform for pathophysiological contexts involving ECM remodeling and mineralization.

Additionally, cells were frequently surrounded by microsized spheroidal structures (green arrows in Figure 4C,D). These features closely resemble those observed in our previous study involving osteoblast precursor cells cultured under similar conditions,^24^ suggesting the presence of matrix vesicles or early mineral deposits. Their spatial association with the cytoskeleton suggests active secretion of mineralizing components, supporting the hypothesis that MOVAS underwent phenotypic transdifferentiation toward a calcifying, osteochondroblast-like phenotype (Figure 4C,D).

Furthermore, the inclusion of κ-Carr maintained scaffold compaction and fibrillar integrity while still allowing robust cell adhesion, morphological adaptation, and potential matrix mineralization (Figure 4C,D). This is particularly relevant considering the structural and functional similarity between κ-Carr and native GAGs, such as dermatan sulfate, which contributes to ECM-cell interactions in vascular tissues. Enhanced interaction with the ECM may favor cytoskeletal reorganization and promote osteogenic signaling cascades. While further spectroscopic or thermogravimetric analyses are needed to confirm the mineral phase identity, the SEM findings strongly support the bioactivity and functional relevance of the scaffold microenvironment in promoting calcification.

### 3.2 Ability of MOVAS to Mineralize a Biomimetic ECM In Vitro

The mineralization capability of MOVAS cells was previously assessed in a 2D environment supplemented with AA and β-GP.^5,6,10,52^ For this, Alizarin Red S staining, a standard method used to quantify matrix mineralization by cells, was employed.^44,53^ After 21 days of culture (Figure 5A), the reddish structures observed in the optical microscopy images of Alizarin Red S-stained samples attested extensive calcium deposition, confirming the transdifferentiation of MOVAS toward an osteochondroblast-like phenotype.

**Figure 5:**
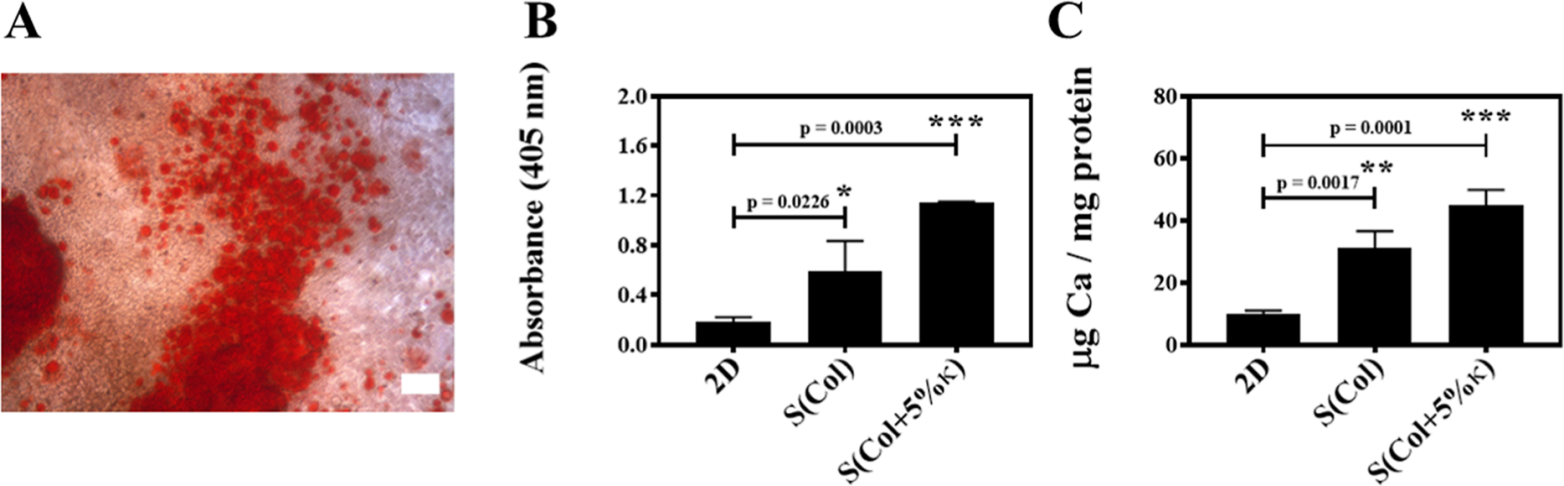
Effect of scaffold composition on matrix mineralization by MOVAS cells cultured under osteogenic conditions for 21 days. (A) Representative image of calcium phosphate nodules stained with Alizarin Red S in the 2D control conditions (polystyrene surface). Microscopy was performed using a Zeiss Axiovert light microscope at 100× magnification. Scale bar: 100 μm. (B) Quantification of calcium deposition by the absorbance at 405 nm. Values were normalized by subtracting the background absorbance from stained but nonmineralized scaffolds, as described in Zhang et al.^43^ (C) Total calcium content normalized to protein concentration (μg Ca/mg protein), determined by colorimetric assay. Data are presented as the mean ± SD from three independent experiments performed in triplicate. Statistical analysis was conducted using one-way ANOVA.

Representative images of the 3D conditions [S­(Col) and S­(Col +5% κ)] are not shown since Alizarin Red S nonspecifically binds to collagen, resulting in uniform background staining across the scaffold surface. This technical limitation, also reported in the literature, impairs morphological interpretation of mineralization when using collagen-based matrices.^43^ To overcome this, the extent of mineralization was quantitatively assessed through absorbance measurements after dye extraction.

The mineralization ability of MOVAS cells seeded on S­(Col) and S­(Col +5% κ) was quantified photometrically by dissolving the bound Alizarin Red S (Figure 5B). Results indicated that seeding MOVAS cells for 21 days on biomimetic 3D scaffolds, the presence of 5 wt % κ-Carr [S­(Col+5%κ)] in the composition of Col-based scaffolds enhanced the mineralization of the ECM by MOVAS cells compared to both 2D cultures (control) and cells seeded on the 3D scaffolds devoid of the polysaccharide [S­(Col)].

The amount of calcium present in the samples relative to the protein contents was higher after seeding cells on scaffolds for 21 days (Figure 5C) than the ratio observed in 2D (control). This result indicates a significant increase in ECM mineralization, particularly in scaffolds containing κ-Carr, and corroborates the Alizarin Red S staining findings (Figure 5B). Notably, this same collagen/κ-Carr scaffold system has previously been shown to exhibit increased mechanical stiffness upon mineralization, as demonstrated by puncture testing with osteoblast-like cells.^24^ This supports the hypothesis that the observed mineral accumulation may also result in enhanced mechanical properties, an established hallmark of pathological calcification.

TNAP activity serves as a marker for calcifying cells, preceding pathological calcifications.^6,9,10^ The ability of MOVAS cells seeded on Col-based scaffolds to mineralize was initially assessed by measuring TNAP activity after 21 days of culture. Cells cultured on scaffolds containing κ-Carr [S­(Col+5%κ)] exhibited significantly higher TNAP activity compared to both the 2D condition (coverslip) and the Col-based scaffolds [S­(Col)] (Figure 6A).

**Figure 6:**
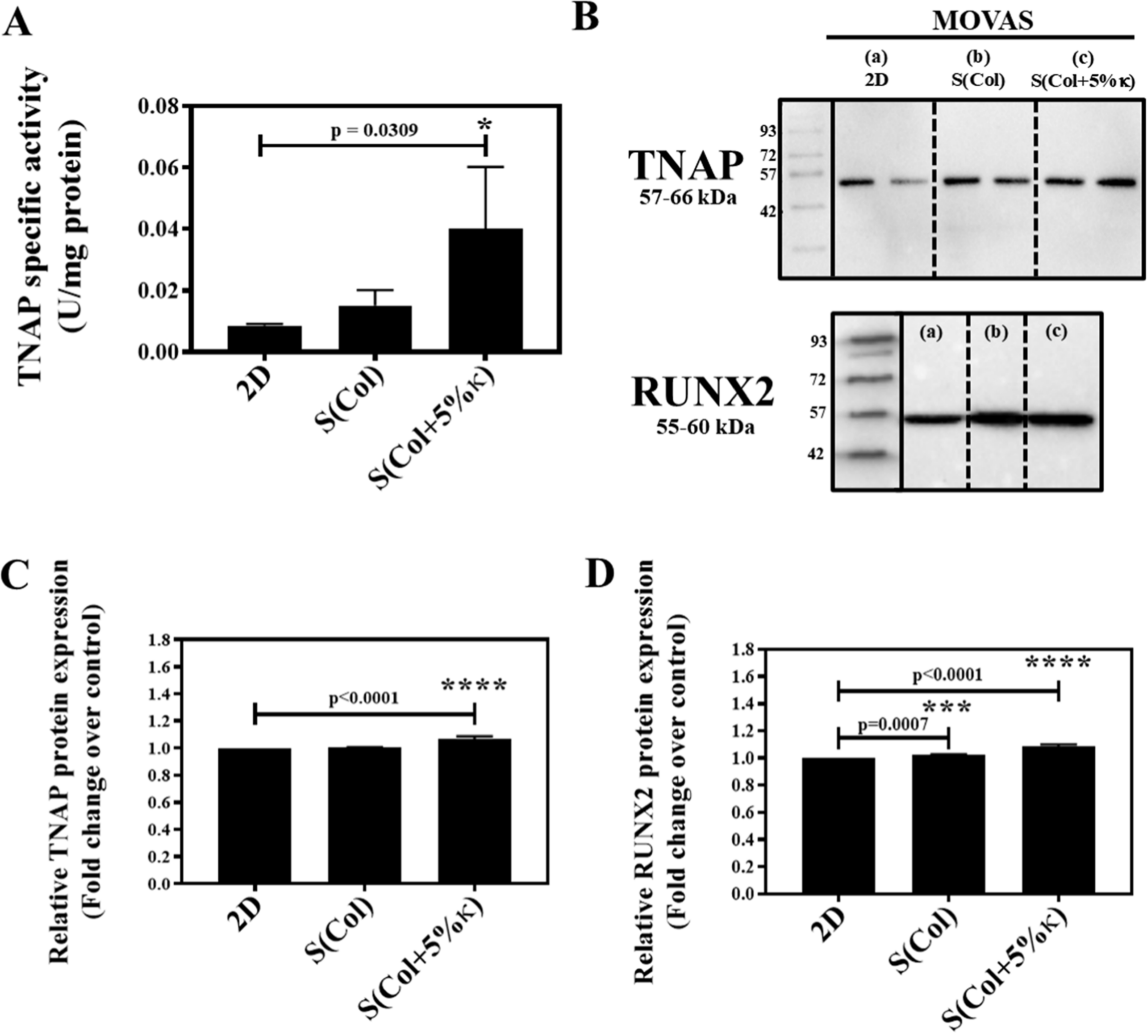
Effect of biomimetic collagen/κ-carrageenan scaffolds on MOVAS cell transdifferentiation under osteogenic stimulation. (A) TNAP-specific enzymatic activity measured in cells cultured for 21 days in 2D (control) and Col-based scaffolds without [S­(Col)] and with incorporation of 5 wt % κ-Carr [S­(Col+5% κ)]. (B) Cropped Western blot images of the TNAP and RUNX2 protein expression. Equal amounts of total protein were loaded per lane (quantified via BCA assay), and a technical duplicate was included for TNAP. (C,D) Densitometric quantification of protein bands using ImageJ, normalized to the 2D control (set to 1). Data are expressed as the mean ± SD from three independent experiments, each performed in triplicate. Statistical significance was determined by one-way ANOVA. Full-length, uncropped membranes (including a biological replicate and Ponceau-stained membrane) are provided in the Supporting Information (Figures S1 and S2).

TNAP is crucial for the mineralization process, and its overexpression can stimulate the deposition of calcium phosphate in the ECM mineralization.^5,6^ To validate the upregulation at the protein level, Western blotting analysis using an antibody specific for the detection of endogenous TNAP was performed. As shown in the Figure 6B, a protein with a molecular weight of around 60 kDa was detected. Therefore, the gels performed on proteins extracted from MOVAS transdifferentiated toward an osteochondroblast-like phenotype displayed strong bands related to TNAP expression, especially when cells were seeded on a 3D biomimetic microenvironment, as indicated in both Figure 6B,C.

Full-length, uncropped Western blot membranes are available in the Supporting Information (Figures S1 and S2), including molecular weight markers, a technical duplicate for TNAP, and an independent biological replicate for both TNAP and RUNX2. Protein loading was normalized based on the BCA assay, and membrane transfer was verified by Ponceau S staining (Figure S3). These complementary measures strengthen confidence in the biological significance of the protein expression differences observed and reinforce the scaffold’s capacity to modulate MOVAS phenotype through combined structural and biochemical cues.^44,47^

The transdifferentiation process can be characterized by the overexpression of osteogenic markers, such as RUNX2, a key transcription factor associated with osteoblast differentiation.^10,54^ The cultivation of MOVAS in an osteogenic medium along with seeding on biomimetic scaffolds resulted in RUNX2 upregulation, compared to cell cultures in 2D and on scaffolds without κ-Carr [S­(Col)], as shown by Western blot analysis (Figure 6B,D).^10,54^ These findings highlighted the importance of the 3D biochemical microenvironment in promoting transdifferentiation toward an osteochondroblast-like phenotype.

Although this study did not investigate upstream signaling pathways such as Wnt/β-catenin or BMP signaling, the increased expression of RUNX2 and TNAP observed in our model reflects the activation of osteogenic programs that are well-established downstream targets of these pathways. Previous studies have shown that both Wnt and BMP signaling converge on RUNX2 and TNAP during vascular calcification processes.^5,55–57^ For instance, Alesutan et al.^55^ demonstrated that periostin enhances calcification via β-catenin signaling, upregulating these markers, whereas Zhu et al.^56^ showed that nesfatin-1 acts through BMP-2 to achieve similar effects. Although these pathways were not dissected here, our findings suggest that the biochemical and structural properties of the κ-Carr-enriched scaffold provide sufficient cues to induce osteogenic transdifferentiation, supporting its potential as a robust proof-of-concept model.^57^

To isolate the effects of scaffold composition on the VSMC behavior, we employed a monoculture of MOVAS cells under osteogenic stimulation. This proof-of-concept approach was chosen to eliminate confounding paracrine signals from other cell types and focus on direct matrix–cell interactions. Similar strategies have been used successfully to study the osteogenic effects of soluble factors or ECM components in VSMC cultures.^5,55,56,58^ In line with those findings, our model showed increased RUNX2 and TNAP expression as well as matrix-bound calcium deposition, confirming the system’s ability to replicate key features of pathological calcification. Although coculture systems offer greater physiological relevance, particularly for studying interactions with endothelial or immune cells, we consider this monoculture-based system a valuable starting point for more complex multicellular models.

Moreover, the scaffold composition, designed to replicate essential ECM components, appears to actively promote MOVAS transdifferentiation. The importance of GAGs in this process has been well documented.^21,23,24^ Herein, we attribute this effect to the sulfated nature of κ-Carr, which structurally resembles dermatan sulfate, a vascular GAG involved in interactions with collagens, growth factors, and heparin cofactor II.^25^ In addition, sulfated polysaccharides may play a role in promoting ECM mineralization. For instance, heparan sulfate has been considered as a receptor to anchor matrix vesicles, released by mineralizing cells, at collagen nucleation sites for apatite formation.^5,46,59–61^ Altogether, these results underscore the relevance of the scaffold composition in directing the MOVAS fate and mineralization, reinforcing the rationale for this bioinspired model as a platform for mechanistic and therapeutic studies of soft tissue calcification.

### 3.3 Characterization of Minerals Produced after Transdifferentiation in a Biomimetic ECM

Thermogravimetric analysis (TGA) was carried out to quantify the mineral formation in the biomimetic ECM. TGA curves revealed that biomimetic Col-based scaffolds, without [S­(Col)] and with incorporation of 5 wt % κ-Carr [S­(Col + 5% κ)] followed a multistage decomposition characterized by three endothermic processes (Figure 7) like Col in bone and dentine.^62–64^

**Figure 7:**
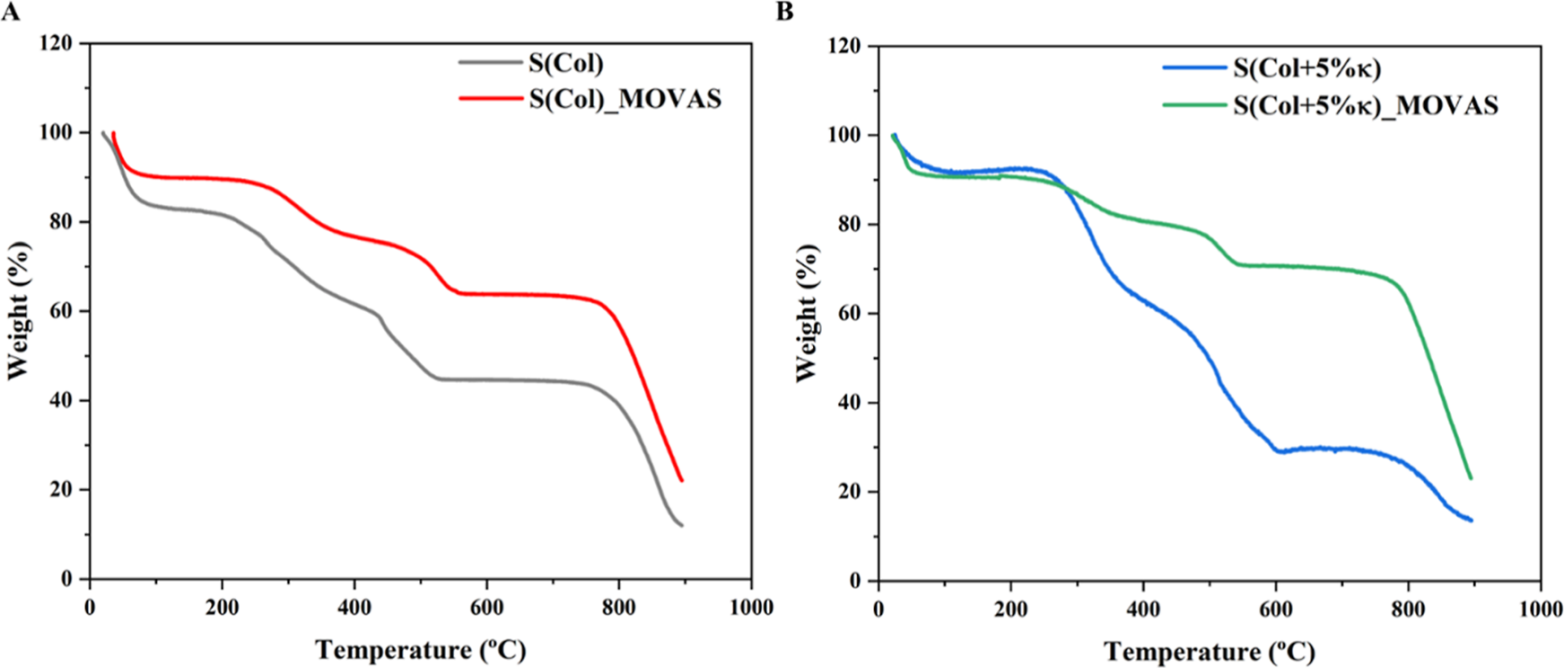
Thermal Analysis and quantification of the mineral formation in biomimetic scaffolds after cell transdifferentiation toward an osteochondroblast-like phenotype. Representative curves obtained by thermogravimetric (TGA) analysis of the 3D Col-based scaffolds without (A) [S­(Col)] and with (B) 5 wt % κ-Carr [S­(Col + 5% κ)] before and after the cultivation of MOVAS cells for 21 days. The mineral content (wt %) of the scaffolds was determined by the difference in ash content at 890 °C before and after seeding MOVAS cells.

The initial weight loss observed in the TGA curves (Figure 7) between 30 and 170 °C is attributed to the release of physiosorbed H_2_O molecules.^62,65^ Following this, the breakdown of Col fibrils occurs in the temperature range between 200 and 470 °C, involving the release of structural water and the generation of products with low molecular weight.^62,64,65^ Subsequently, the combustion of residual organic components, between 450 and 800 °C, culminates in the total decomposition of the organic matrix.^48,62,64,65^ The quantification of the mineral content in the matrices can be calculated by the relative mass remaining after heating up to 890 °C.^64,65^ A concise presentation of transition temperatures and their corresponding percentage of weight loss is available in Table 1.

**Table 1:** Peak Temperature (*T*) and Weight Losses (*W* %) Related to Thermal Processes Observed in TG-DGT Plots before and after Mineralization Induced by MOVAS on Scaffolds^a^

sample	initial weight	peak		final weight	mineral content
	*W*_i_ (mg)	*T* (°C)	*W*_890_ (%)	*W*_f_ (mg)	*W*Δ before and after MOVAS cultivation
S(Col)	0.578	890	12	0.069	0.634
S(Col)_MOVAS	3.194	890	22	0.703	
S(Col + 5% κ)	0.482	890	10	0.048	0.697
S(Col + 5% κ)_MOVAS	2.692	890	24	0.646	

a The mineral content (*W*Δ) in the scaffolds was determined by the difference in ash content at 890°C before and after cell seeding.

Considering the complete removal of adsorbed water at 180 °C, the weight difference of the scaffolds before and after cultivation of MOVAS in osteogenic medium for 21 days was calculated after heating to 890 °C. The scaffolds containing MOVAS resulted in an increase of the final weight after cultivation, which can be assigned to the mineral deposition stimulated by the cells. Moreover, the addition of κ-Carr to the scaffolds stimulated mineral formation. The final weight increased up to 10% for MOVAS cultivated in the presence of κ-Carr compared with the Col-based scaffolds [S­(Col)]. Consistent with our previous work,^24^ the incorporation of κ-Carr to the Col-based scaffolds stimulates the activity of osteoblasts without compromising cell viability. Corroborating the results obtained from TNAP activity, Alizarin Red staining, and calcium quantification, the importance of exploring the role of the sulfated polysaccharide can be underscored in mimicking the function of naturally occurring GAGs in the calcification process.^24,30,50,66^ Furthermore, the significant contribution of the polysaccharide to mineralization is noteworthy since samples containing κ-Carr exhibited the highest mineral content. Although the osteogenic potential of κ-Carr has been reported,^24,50,66^ the pathways and mechanisms involved during the differentiation process in calcifying phenotype and further mineralization are still unclear.

The chemical composition of the mineral phase deposited in the biomimetic Col-based scaffolds during MOVAS transdifferentiation toward an osteochondroblast-like phenotype was investigated. Initially, the minerals formed by these cells were examined in both 2D and in the biomimetic Col-based scaffolds without [S­(Col)] and with 5 wt % κ-Carr [S­(Col + 5% κ)] using Fourier-transform infrared spectroscopy (FTIR) and Raman spectroscopy.

Biological apatite, a calcium phosphate that resembles the chemical composition and structure of hydroxyapatite [Ca_10_(PO_4_)_6_(OH)_2_] (HAp), is typically present in mineralized tissues.^67^ Typical substitutions of OH sites and PO_4_^3−^ sites by carbonate ions are named type A and type B, respectively.^67,68^ Biological apatite found in the mineralized tissues of mammals is found in a partially carbonated form.^67,68^

FITR spectra were recorded before and after the cultivation of MOVAS for 21 days in 2D and both scaffolds in osteogenic medium. FTIR spectra of the minerals (Figure 8A) display the characteristic bands at 1091, 1020, and 961 cm^−1^ related to the phosphate (PO_4_^3−^) and at 3571 cm^−1^ to hydroxyl (OH^−^) groups.^68^ The bands close to 2900−2800 and 1600−1500 cm^−1^ can be assigned to the presence of organic compounds after the cultivation of cells in 2D. Due to the composition of the scaffolds that give rise to several bands in the FTIR spectra, the direct identification of apatite formation after cell cultivation in 3D was challenging (Figure 8A). Bands corresponding to organic components of mineralized tissue, such as collagen, were observed. For instance, bands at 1652 and 1550 cm^−1^ can be identified as amide I (C=O stretching mode) and amide II (N–H bending and C–N stretching modes).^68^ Other bands at 2919, 2851, and 3291 cm^−1^, assigned to C–H, N–H, and O–H stretching, could be observed.^68^ The addition of κ-Carr to the scaffolds seemed to favor the formation of apatite. After cultivation of MOVAS for 21 days, an increase in the intensity of the band around 1020 cm^−1^ can be observed for the scaffolds containing the κ-Carr, compared to both cells seeded on 2D and the Col-based scaffolds (Figure 8A, black, red, and purple lines, respectively).

**Figure 8:**
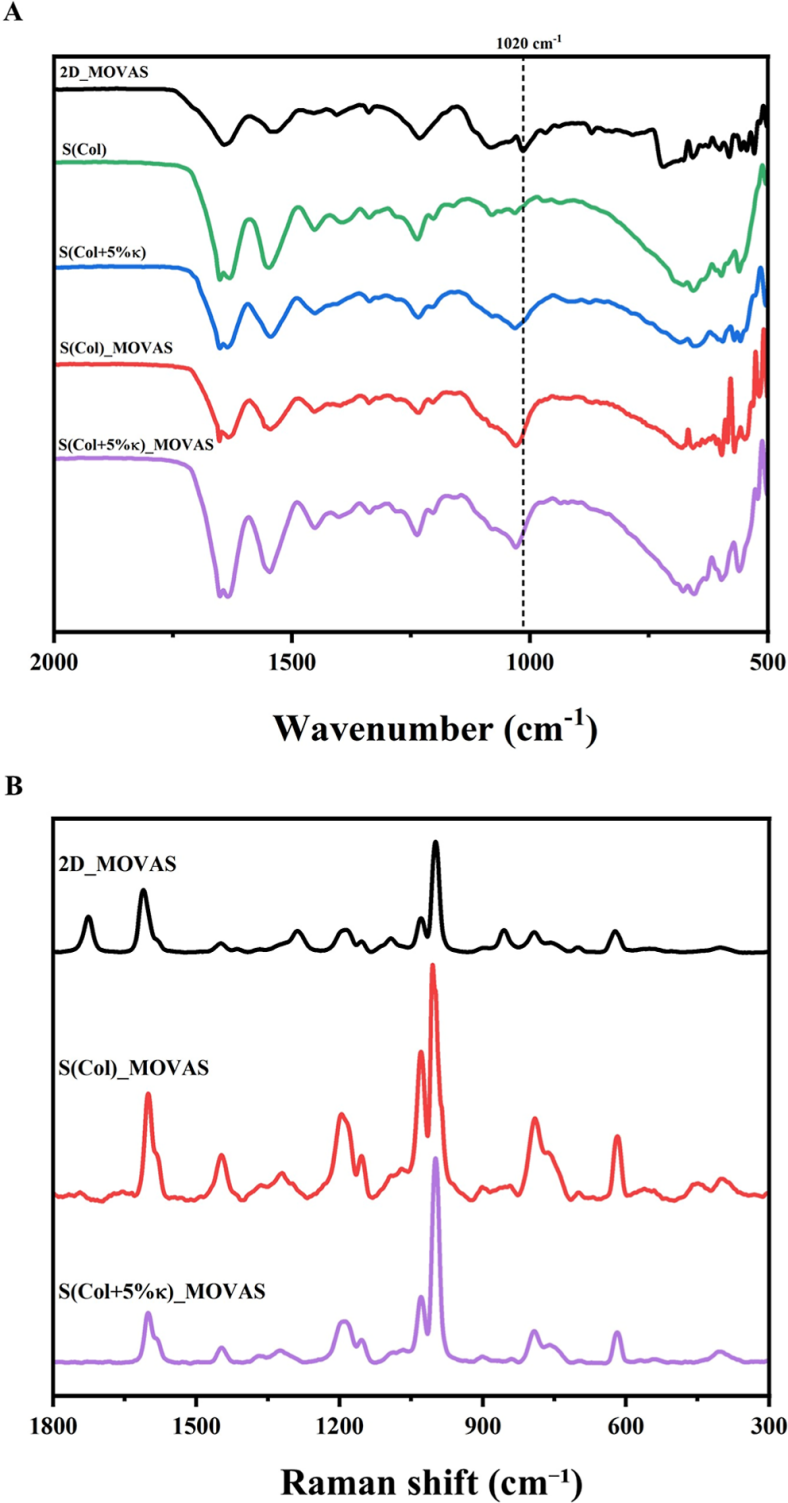
Chemical characterization of the mineral precipitated by seeded MOVAS cells. (A) Representative spectra obtained by FTIR before and after the MOVAS culture for 21 days on polystyrene coverslip (2D) (black line) and on the Col-based scaffolds without [S­(Col), green and red lines, respectively] and with the incorporation of 5 wt % κ-Carr [S­(Col + 5% κ), blue and purple lines]; *n* = 3 ± SEM. (B) Representative spectra obtained by Raman of MOVAS cells cultured on 2D (black line) and on both scaffolds, S­(Col) (red line) and S­(Col + 5% κ) (purple line) for 21 days; *n* = 3 ± SEM.

In this perspective, Raman spectroscopy was employed to gather detailed information on both mineral content and organic matrix components. For example, the highly intense band near ∼960 cm^−1^ (vibrational modes of PO_4_^3−^) is characteristic of carbonated apatite or HAp and is the most used marker for bone and calcified tissues.^69–73^ Regarding the organic matrix, the presence of bands in the regions of 1700−1100 and 900−700 cm^−1^ can be attributed to organic compounds. For example, peaks around 1241−1269 (amide III), 1670 (amide I) cm^−1^ can be attributed to amide bonds in collagen.^69^ Additionally, bands around 1063 cm^−1^ assigned to the vibrational modes of S=O and 1375−1410 cm^−1^, assigned to (COO−) and pyranose structure of κ-Carr, can be observed.^69^

Analyzing the spectra obtained after the cultivation of MOVAS in both 2D and Col-based scaffolds, without [S­(Col)] and with 5 wt % κ-Carr [S­(Col + 5% κ)] (Figure 8B), an intense band can be observed close to ∼970−1015 cm^−1^, with a maximum around 990 cm^−1^. The specific Raman signature does not match the Raman bands assigned to any crystalline calcium phosphate phases: octocalcium phosphate (OCP) at 960−962 cm^−1^, brushite at 990 cm^−1^, and HAp at 962−963 cm^−1^ and aqueous species (H_2_PO_4_^−^ at 880 and 1080 cm^−1^, HPO_4_^2−^ at 992−993 cm^−1^, and PO_4_^3−^ at 939 cm^−1^).^69^ However, it is important to realize that bone mineral is not a pure stoichiometric compound, and slight displacement in the observed frequencies may occur.^70^ Raman spectroscopy can be used to investigate the maturation of the mineral from a transient phase (OCP-like) to a more stable phase (carbonated apatite-like) related to the interference of the organic matrix. Moreover, the displacement in the frequency can be used for the identification of transient intermediates formed under physiological and pathological conditions.^70–73^ For example, in some calvaria samples, bands at 980−985 cm^−1^ and sometimes an OCP band at 1010−1014 cm^−1^, which are Raman signatures for transient intermediates that coexist in the very early phase of mineralization, can be observed.^70^ The presence of a monohydrogen phosphate P–O stretching at 980−985 cm^−1^ suggests that other phosphate phases besides OCP and amorphous calcium phosphate (ACP) may be transiently present, such as dicalcium phosphate dihydrate which presents a band at 985 cm^−1^.^70^ Analyzing the Raman spectra shown in Figure 8B, the peak around 970−1015 cm^−1^ can be attributed both to the formation of brushite, a stable phase of calcium phosphate found in calcified tissues, and to the presence of transient intermediates, like OCP, or aqueous species, as dicalcium phosphate dihydrate.^70,72,73^

The semiquantitative X-ray energy dispersive analysis (EDS) was used for elemental identification of the minerals formed after cell cultivation in 2D and on both Col-based scaffolds, without [S­(Col)] and with 5 wt % κ-Carr [S­(Col + 5% κ)] (Figure 9). The simultaneous presence of peaks related to P at 2 keV and to Ca between 3.5 and 4 keV was observed only in the spectra obtained for samples after MOVAS cultivation with exogenous osteogenic supplementation (Figure 9B,D,E). The molar Ca/P ratio was calculated from the spectra. The values obtained for the minerals deposited from cells cultivated in 2D and both scaffolds, S­(Col) and S­(Col + 5% κ), are in the 1.40–2.00 range, as shown in Table 2. The Ca/P ratio of 1.67 assigned to the structure of stoichiometric HAp does not match the Ca/P ratio of biological apatite, due to cationic and anionic substitutions.^5,67^ The typical chemical composition of biological apatite reported in the literature is Ca_8.3−1.7_(PO_4_)_4.3_(HPO_4_ or CO_3_)_1.7_(OH or 1/2 CO_3_)_0.3−1.7_, with a Ca/P molar ratio varying between 1.93 and 1.38.^67,74,75^ In this sense, it is important to highlight that the composition and organization of minerals in calcified tissues widely vary among the tissues, individuals, and aging, translating to different Ca/P ratios during crystal formation and maturation.^67,74,75^

**Figure 9:**
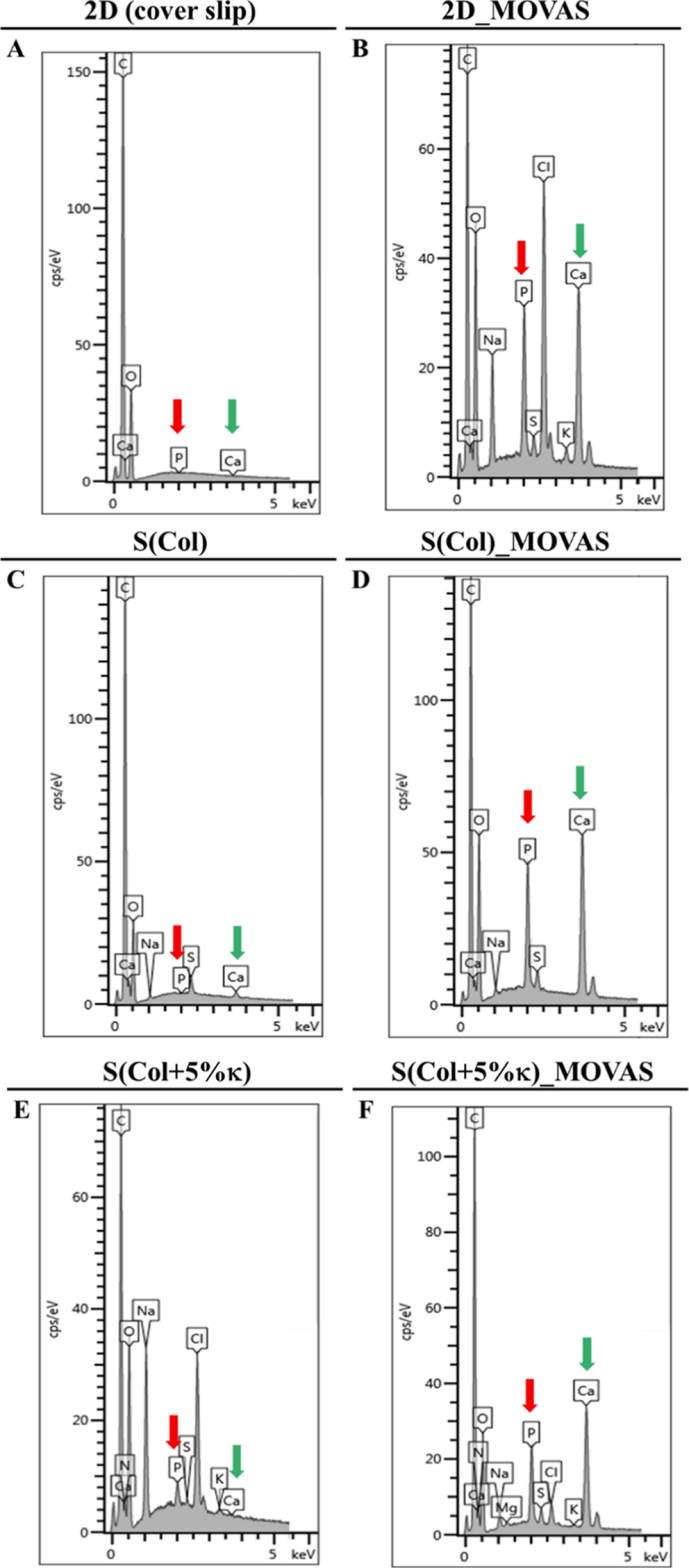
Chemical composition of mineral phase produced by MOVAS transdifferentiated toward osteochondroblast-like cells. Representative spectra obtained by EDS before and after the MOVAS were cultivated for 21 days, with exogenous osteogenic supplementation, on (A,B, respectively) polystyrene coverslip (2D) and on the Col-based scaffolds, (C,D, respectively) without and (E,F, respectively) with the incorporation of 5 wt.% κ-Carr; *n* = 3 ± SEM. The green and red arrows indicate, respectively, the presence of calcium (Ca) and phosphorus (P) related to the formation of calcium phosphate by MOVAS transdifferentiated toward calcifying phenotype.

**Table 2:** Calculation of the Molar Ratio Ca/P from EDS Analysis of the Surfaces of Polystyrene Coverslip (2D) and on the Col-Based Scaffolds, without [S­(Col)] and with the Incorporation of 5 wt % κ-Carr [S­(Col + 5% κ)], before and after the MOVAS Were Cultivated for 21 Days with Exogenous Osteogenic Supplementation

sample	EDS
2D (Coverslip)	0
2D_MOVAS	1.51 ± 0.02
S(Col)	0
S(Col)_MOVAS	1.63 ± 0.04
S(Col + 5% κ)	0
S(Col + 5% κ)_MOVAS	1.93 ± 0.03

The cultivation of cells on both Col-based scaffolds, S­(Col) and S­(Col + 5% κ), led to an increase in the Ca/P molar ratio compared to 2D, as shown in Table 2. Besides the stimulation by the 3D microenvironment, it can be considered that the composition of the biomimetic ECM can influence the formation of the mineral phase resulting from the transdifferentiation of MOVAS toward an osteochondroblast-like cell, acquiring a calcifying phenotype after 21 days of culture with exogenous osteogenic supplementation. For example, scaffolds containing κ-Carr resulted in the higher Ca/P ratio, which must correspond to the presence of stable mineral phases, given the proximity to the ratio reported for native tissues (1.93–1.38).^67,74,75^ The stabilization of mineral phases can come from both calcium deficiency and replacement of either PO_4_^3−^ or OH^−^ ions in the chemical structure of HAp.

Figure 9A,B also shows the presence of carbon (C) and oxygen (O) peaks, which are key elements of the polystyrene coverslip. However, in all spectra obtained from the analysis of the scaffold surface (Figure 9C–F), the presence of intense signals related to C and nitrogen (N) is attributed to key elements constituting the organic matrix. Finally, the peaks of S, Cl, Na, and K might have been generated from the remnants of some components of PBS or Cell Culture Media. Furthermore, for the scaffolds containing 5 wt % κ-carr, the increase in signal related to the S element, mainly after MOVAS cultivation for 21 days, can also be related to the sulfate group present in the structure of this polysaccharide.

Therefore, qualitative evidence of scaffold integrity was supported by the preservation of structures observed through SEM, FTIR, Raman spectroscopy, and EDS, even after 21 days of culture and mineral deposition. This aligns with the broader literature, indicating that early stage models of vascular calcification may not require functional assays when reliable marker expression and matrix mineralization are present.^5,55,56^ The enhanced expression of RUNX2 and TNAP (Figure 6B–D), together with matrix-bound calcium phosphate deposition, supports the notion that the scaffold composition alone can actively modulate the acquisition of a calcifying phenotype by MOVAS cells.

## 4 Conclusion

This study highlights the versatility of 3D Col/κ-Carr-based scaffolds in recapitulating in vitro pathological calcification processes such as vascular calcification. By evaluating the impact of a biomimetic 3D microenvironment and exogenous osteogenic supplementation, we demonstrated that this system effectively supports the osteogenic transdifferentiation of MOVAS into osteochondroblast-like cells, which acquire a calcifying phenotype after 21 days of culture. As a proof-of-concept model, this scaffold system successfully demonstrates that matrix composition alone can influence VSMC transdifferentiation, reinforcing its potential for future mechanistic studies and the development of therapeutic strategies targeting pathological mineralization.

Our findings suggest that the ECM calcification, achieved through the transdifferentiation of MOVAS, can be enhanced by seeding these cells on the 3D scaffold, supplemented with β-glycerophosphate and ascorbic acid. The role of κ-Carr in promoting this transdifferentiation process is evident from the quantification of calcium deposition, TNAP activity, and Alizarin Red staining. Additionally, TGA and spectroscopic techniques, such as EDS and Raman spectroscopy, provided crucial insights into the mineralization process, revealing the formation of a carbonated apatite-like mineral phase, with a Ca/P ratio ranging from 1.38 to 1.93. These findings indicate that the biomimetic 3D environment plays a pivotal role in regulating mineral deposition, potentially favoring the formation of an apatite-deficient structure that deviates from stoichiometric hydroxyapatite (1.66).

This study provides valuable insights into the chemical composition and structural features of mineralized tissue during the investigation of pathological calcification. Furthermore, the model presented here offers a platform for screening regenerative biomaterials and investigating pathological soft tissue mineralization in vitro under both physiological and pathological conditions. Further research into the mechanistic processes of transdifferentiation and mineralization will be essential to develop patient-specific models for testing therapeutic strategies aimed at preventing pathological calcification.

## Supplementary Material



## Notes

Uncropped Western blot membrane and Ponceau S-stained membrane (PDF)
