# Supplementary material for: Bioinspired Collagen/κ-Carrageenan 3D Matrix for In Vitro Modeling of Vascular Calcification
Source: ACS Biomater Sci Eng. 2025 Jul 19;11(8):5012–26. doi: 10.1021/acsbiomaterials.5c00754 (PMC12344643; doi:10.1021/acsbiomaterials.5c00754)
Supplement: Supplementary file 1 [file ab5c00754_si_001.pdf]

A Bioinspired Collagen/κ-Carrageenan 3D Matrix for In Vitro Modeling of Vascular Calcification

L.F.B. Nogueira[1*], M. T. de Melo[1], J. G. Cominal[1], K. R. da Silva[2], S. Y. Fukada[2], M. Bottini[3,4], L. Brizuela[5], P. Ciancaglini[1], S. Mebarek[5], A.P. Ramos[1*]

1-Department of Chemistry, Laboratory of Physical Chemistry of Surfaces and Colloids, Faculty of Philosophy, Science and Letters at Ribeirão Preto, University of São Paulo, 14040-901, Ribeirão Preto-SP, Brazil.

2- Department of BioMolecular Sciences, School of Pharmaceutical Sciences of Ribeirao Preto, University of Sao Paulo, 14040-903, Ribeirão Preto-SP, Brazil.

3- Department of Experimental Medicine, University of Rome Tor Vergata, 00133 Rome, Italy

4- Sanford Burnham Prebys, La Jolla, California 92037, United States

5-Université de Lyon; CNRS; UCBL; UMR 5246 - Institut de Chimie et de Biochimie Moléculaires et Supramoléculaires, 43, Boulevard du 11 Novembre 1918-69622, Villeurbanne, France.

*Corresponding Author:

Lucas Fabrício Bahia Nogueira

Email: lucas.fabricio.nogueira@usp.br

Supporting Information

This Supporting Information includes the full-length, uncropped Western blot membranes corresponding to Figure 6 of the main text. The membranes show TNAP and RUNX2 expression in MOVAS cells cultured in 2D and 3D collagen/κ-carrageenan-based scaffolds under osteogenic conditions. Additional controls include a Ponceau S-stained membrane (to confirm uniform transfer and loading) and an independent biological replicate confirming the reproducibility of the findings.

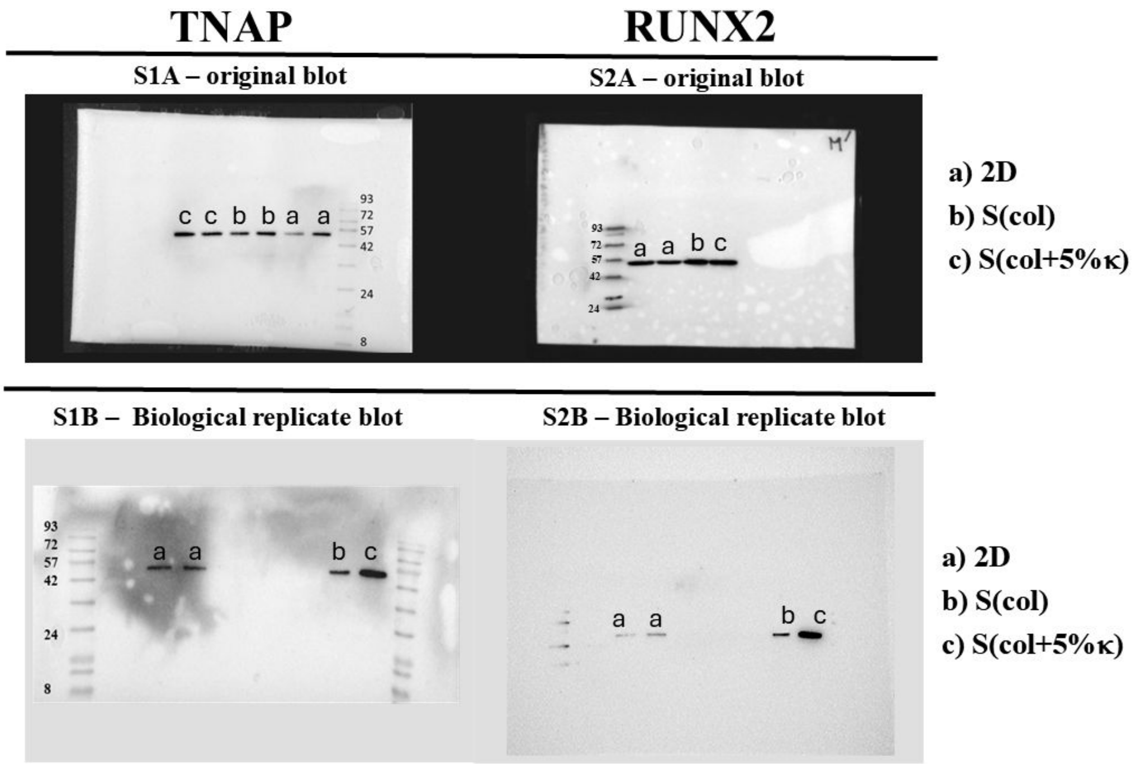

Figure S1. (A) Uncropped Western blot membrane for TNAP expression shown in Figure 6B. (B) Full-length membrane from an independent biological replicate, confirming reproducibility. **Figure S2.** (A) Original uncropped blot for RUNX2 corresponding to the cropped image shown in Figure 6B. (B) Independent biological replicate blot under the same conditions, confirming reproducibility. Lanes: a = 2D (control), b = S(Col), c = S(Col + 5% κ). Molecular weight markers (in kDa) are indicated. Equal protein loading was ensured using BCA quantification. Both membranes were developed under identical ECL exposure settings.

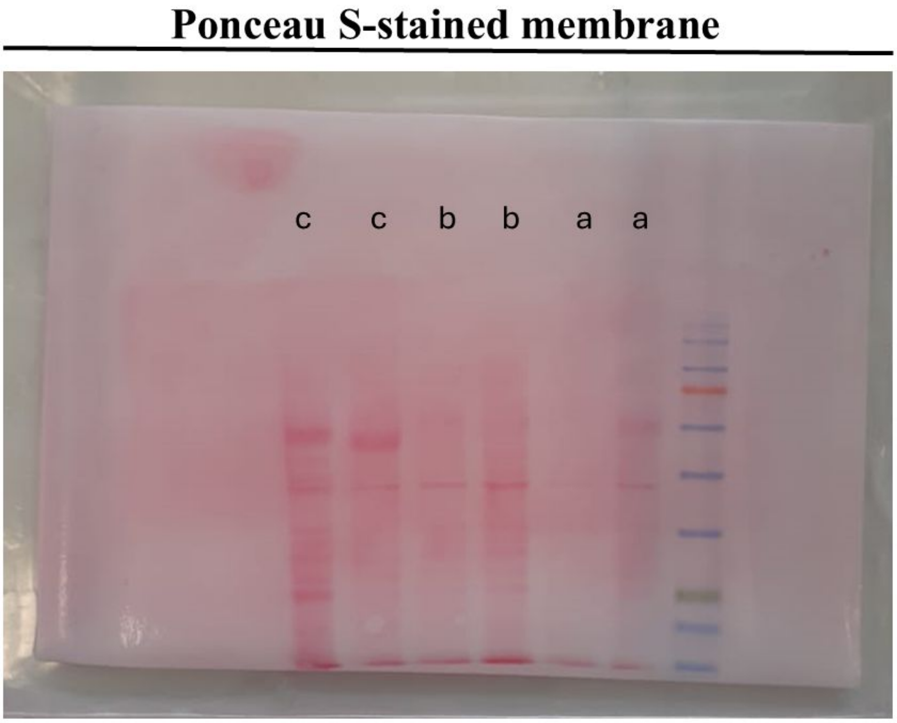

Figure S3. Ponceau S-stained membrane showing total protein from the same experimental conditions (a–c). The staining confirms consistent protein loading and uniform transfer across lanes. This membrane corresponds to the blot shown in Figure S1A.

All Western blot experiments were performed using equal amounts of protein, quantified by BCA assay prior to gel loading. Membranes were imaged under identical exposure conditions using the Chemidoc XRS+ system (Bio-Rad). No digital alterations were applied beyond cropping for presentation in Figure 6. The inclusion of full-length membranes, a technical replicate for TNAP, and a biological replicate for both TNAP and RUNX2 strengthens the reproducibility and reliability of the results.
